# Bioactivity of Bac70 Produced by *Bacillus atrophaeus* Strain DDBCC70

**Published:** 2020

**Authors:** Mohammad Reza Sarjoughian, Shamsozoha Abolmaali, Shakiba Darvish Alipour Astaneh

**Affiliations:** 1. Department of Biotechnology, Faculty of Biotechnology, Semnan University, Semnan, Iran; 2. Department of Biology, Faculty of Basic Sciences, Semnan University, Semnan, Iran

**Keywords:** Antibiofilm, *Bacillus atrophaeus*, Bacteriocin, *Klebsiella pneumoniae*, *Pseudomonas aeruginosa*

## Abstract

**Background:**

Recently, using antibacterial peptides has been considered as a strategy to manage the worldwide antibiotic-resistance crisis. Screening of Dasht-Desert Bacterial Culture Collection (DDBCC) for bacteriocin or bacteriocin-like producer was aimed in this study to introduce native antibacterial agent(s).

**Methods:**

In this study, 170 isolates were examined by the cross-streak method against G+ and G− indicators. Isolates with antimicrobial activity were compared using turbidity and well diffusion tests. The candidate isolate, DDBCC70, was molecularly and biochemically characterized. Then, the production of an antibacterial agent was physicochemically optimized. The supernatant was saturated ammonium sulfate. SDS-PAGE and Thin-Layer Chromatography (TLC) analyses, cytotoxicity, and hemagglutination tests were performed.

**Results:**

First, 23 isolates were detected with antimicrobial activity against at least three of the indicator strains. DDBCC70 was distinguished with the broad-spectrum of antibacterial effects of the Cell-Free Supernatants (CFSs). The black pigments on BHI and a 98% similarity in 16S rDNA and similarity in biochemical tests confirmed the strain of DDBCC70 as *Bacillus atrophaeus (B. atrophaeus)*. The highest amount of the antibacterial agent, Bac70, was obtained from the modified brain heart infusion medium. It was revealed that 70% ammonium sulfate-saturated Bac70 was 3.8 and 1.6 times more effective on *Pseudomonas aeuroginosa* *(P. aeuroginosa)* and *Klebsiella pneumoniae (K. pneumoniae)*. Bac70, a >25 *kDa* protein and a safe compound for blood cells, neither agglutinated human erythrocyte nor lysed sheep blood. The purified bacteriocin-like molecule destroyed biofilms from *P. aeruginosa* and *Staphylococcus aureus (S. aureus)*. Moreover, the fraction of Bac70 from the TLC plate showed higher inhibitory effects against *K. pneumoniae*.

**Conclusion:**

Based on the above-mentioned features, Bac70 is a potential alternative therapeutic agent in pharmaceutical, food preservative and biotech-related industries.

## Introduction

Antibiotic resistance, a serious problem in hospital and food industries, has encouraged researchers to develop new strategies *via* definitive treatments and substitutions. The most promising strategy has been discovering new antibacterial agents from natural resources with wide or narrow and specific bacterial spectra ^1^. Natural bioactive molecules, mainly secondary metabolites from bacteria and fungi, have largely contributed to the pharmaceutical industry due to their structural and functional diversity ^2^.

Bacteriocins are antibacterial peptides synthesized in bacteria either by ribosomes and/or NonRibosomal Peptide Synthetase (NRPS) enzymes. While the producer bacteria are protected by specific immunity proteins, bacteriocins cause the death of related or non-related bacteria or may inhibit them. Bacteriocins target bacterial cell wall and play a key role in bacterial communication with a specific and efficient antagonistic activity ^3,4^. These proteins/peptides are diverse in their structure and function as well as the mode of action. Therefore, a broad range of bacteria are targeted by bacteriocins under different environmental conditions. These features make them suitable solution for overcoming the antibiotic resistance problem in food and healthcare industry. Bacteriocins are produced naturally or transgenically ^5^.

Variations in biochemical properties, molecular weight, the spectrum of activity, low toxicity, antibacterial function in the closely-related species, and the potential for bioengineering have made bacteriocins outstanding antibacterial agents ^6^. Moreover, bacteriocins act specifically based on the cell wall differences naturally occurred between the two groups of bacteria, G+ and G^−^ 7. On the other hand, the superior feature of bacteriocins is biofilm destruction and inhibitory effects on biofilm formation.

The potential for producing bioactive molecules varies structurally and functionally in different microbiomes ^8^. The native antibacterial agent might be more efficient against antibiotic-resistant bacteria and for disruption of biofilm.

This study was aimed to screen Dasht-Desert Bacterial Culture Collection (DDBCC) for valuable bacteriocin (s) or bacteriocin-like molecules. The collection includes the isolates from Haj-AliGholi-Khan salt lake, the north of Dasht desert, Semnan, Iran. It was supposed that the microbiome of Haj-AliGholi-Khan salt lake most probably produces biomolecules including peptides or small proteins to win the surviving competition in this extreme environment. Literature supported the idea and up to our knowledge, this is the first study on the microbiome of Haj-AliGholi-Khan salt lake for bacteriocins.

## Materials and Methods

### Ethical statement

Whole blood was taken from healthy volunteers with informed consent. The study was conducted according to ethical principles of the Declaration of Helsinki 1964 and its later amendments and comparable ethical standards.

### Bacterial strains and screening DDBCC against indicators

The antibacterial activities of 170 strains from Dasht-Desert Bacteria Culture Collection (DDBCC) were examined against *Bacillus cereus (B. cereus)* (PTCC1015), *Escherichia coli (E. coli)* (ATCC25922), *Bacillus subtilis (B. subtilis)* (ATCC12711), *Pseudomonas aeruginosa (P. aeruginosa)* (ATCC27853), *Salmonella* (spp.), *Proteus* (spp.), *Klebsiella pneumoniae (K. pneumoniae)* (ATCC13883) and *Staphylococcus aureus (S. aureus)* (ATCC25923) using cross-streak assay on nutrient agar ^9^. The isolates were re-streaked on nutrient agar plate.

### Well diffusion and bactericidal assay

Based on the results from the cross-streak assay, 23 out of 170 strains were tested for their antibacterial activity using well diffusion agar method. The isolates were inoculated (1%) into Brain Heart Infusion (BHI, Merck) broth and incubated for 24 *hr* at 30°*C*, 130 *rpm*. The supernatant was precipitated, and then filtered (0.45 *μm*, Merck). Cell-Free Supernatants (CFS; pH=7.0) were sterilized with a 0.45 *μm* membrane. Next, 200 *μl* of the supernatant was applied to the wells on BHI agar, inoculated with each indicator bacterium, and incubated at 37°*C* for 24 *hr*.

The strains with the highest inhibition zone were cultured in BHI broth for 24 *hr* at 37°*C*. The indicator strains with a turbidity of 0.5 McFarland were treated by the CFS at a ratio of 1:10. The growth rates of the indicators were measured for 24 *hr* (interval of 2 *hr*). DDBCC70 was identified with a broad spectrum of antibacterial activity ^10^.

### Optimization of physicochemical conditions

The effects of 10% (*v/v*) of chloroform, isopropanol, diethyl ether, methanol, 1-butanol, ethyl acetate, dichloromethane, carbon tetrachloride, ethanol, acetone, DMSO, inorganic salts, 5 *mM* of FeSO_4_, MgSO_4_, NiSO_4_, CaSO_4_, MgCl_2_, KCl, NaCl, NiCd and the detergents of urea, SDS, EDTA and 1% (*v/v*) Triton X-100 were examined on the CFS. The antibacterial activity of the CFS was determined using the well diffusion method against the indicators (all the materials were Merck products).

DDBCC70 was cultured in BHI supplemented with 0.5, 1, 1.5 and 2% (*w/v*) of tryptone, peptone and yeast extract (Merck) as the nitrogen resource, followed by incubation for 24 *hr* at 37°*C*. The same experiments were done in M9 medium supplemented with 1% glucose, mannitol, sucrose, starch, carbonate-calcium and/or carbonate-sodium for optimizing carbon resource ^11^. All the experiments were performed in triplicate.

DDBCC70 was incubated in the modified BHI (with 1% glucose and peptone) for 12, 24, 36 and 72 *hr* at 37°*C*. The CFS from DDBCC70 culture was subjected to a range of pH from 3 to 11 using 1N HCl and 1N NaOH, incubated for 5 *hr* at 25°*C* and pH readjusted to 7. The CFS was incubated at 60–100°*C* by a step of 10°*C* for 15, 30, 45 and 60 *min*, at 121°*C* for 15 *min*, at −20, −40 and −70°*C* for 30 *min*. To test UV irradiation effects, the CFS was exposed to UV (360 *nm*) for 15 *min* ^12,13^.

### Partial purification of bacteriocin-like Bac70

Bac70 from the CFS was partially purified at 20, 40, 50, 60 and 70% ammonium sulfate (Merck) saturation for 16 *hr* at 4°*C* ^14^. The proteins were isolated at 8000 *g*, 4°*C* for 45 *min*, dissolved in 50 *mM* PBS buffer, and dialyzed (3 *kDa*) against the same buffer for 24 *hr* at 4°*C*.

### Determination of MIC of Bac70

The final concentrations of Bac70 were progressively halved to 500, 250 and 125 to 8 *μg/ml* in the 96-well plate. Next, 5 *μl* of 10^5^ *CFU/ml* of each indicator was seeded into the well containing a certain amount of Bac70 and incubated for 18 *hr* at 37°*C*. From the well, indicating Minimum Inhibitory Concentration (MIC) value, 10 *μl* was plated onto BHI and incubated for 16 *hr* at 37°*C*. The Activity Unit (AU) for Bac70 was calculated as:

$$\mathrm{AU}/\mathrm{ml}=2^{\text{n}}\times 1000/200^{14}$$

where “n” is the MIC.

### Qualitative analysis of Bac70

The CFS of DDBCC70 was treated with 1 *mg/ml* proteinase K for 4 *hr* at 37°*C* followed by the well diffusion test to confirm the nature of Bac70. Thin-layer chromatography was performed for the purified and non-purified Bac70 on TLC silica gel 60 (TLC-plate, Merck Millipore) using butanol, acetic acid and H_2_O (20:10:40) as a solvent system. The spots were visualized by ninhydrin treatment, collected, dissolved in tris buffer, and tested for antibacterial activity against the indicators. The non-purified Bac70 was analyzed on 12% polyacrylamide SDS-gel and stained with Coomassie blue R250 ^9,15,16^. The amount of Bac70 was calculated via Bradford’s assay and a standard curve of 0.02–1 *mg/ml* BSA^17^.

### Cytotoxicity assay

MTT assays were done with the lymphocytes collected by ficoll density gradient. The cells (5×10^4^) were seeded in 100 *μl* of the DMEM medium (Bio- Sera) supplemented with 10% FBS (DNABiotech) and treated with 1479 *AU/ml* of Bac70 for 16 *hr* at 37°*C*. MTT (DNABiotech) at a final concentration of 10% was added for 4 *hr*. The OD_570_ of formazon in 100 *μl* of DMSO (Sigma) was measured ^18^. The experiments were carried out in duplicate.

### Hemagglutination and hemolytic activity

Human red blood cells were washed with PBS, re-suspended in PBS (10%), mixed with 1479 *AU/ml* of Bac70 for 1 *hr* at 4°*C*, and analyzed against PBS as a negative control. DDBCC70 was cultured on sheep blood agar for 18 *hr* at 37°*C* to test hemolytic activity ^19^.

### Biofilms disruption by Bac70

For biofilm formation, the overnight cultures of *S. aureus* and *P. aeruginosa* (10 *μl*) were inoculated in 240 *μl* LB for 7 days at 37°*C* in a 96-well plate. The cultures were removed, and the wells rinsed several times with tris buffer (10 *mM*, pH=7). The purified Bac70 (280 *μl*) from DDBCC70 was transferred into the wells and incubated for 3 *hr* at 30°*C*. The supernatant was removed, the wells were washed several times with tris buffer, filled with 280 *μl* of crystal violet (2%) for 5 *min*, and washed with tris buffer. The OD570 was measured after adding 280 *μl* of acetic acid to the wells. The percentage of biofilm disruption was calculated as ^20^:

$$\text{Disruption}\;\%=\left(\left[{\text{OD}}_{570}\;\text{control}-{\text{OD}}_{570}\;\text{test}\right]/{\text{OD}}_{570}\;nm\;\text{control}\right)\times 100$$

### Bac 70 and penicillin synergy

First, 100 *μl* of *Proteus* (spp) culture (0.5 McFarland) was seeded on nutrient agar followed by making wells on the plate. Then, 100 *μl* of the purified Bac70 and a 100-*μl* mixture of the purified Bac70: penicillin G in a ratio of 1:1 (*v/v*) were dispensed into the wells, and incubated at 37°*C* for 24 *hr*.

### Antibacterial activity assay

Activity Unit of the bacteriocin (AU) was calculated using this formula ^21^:

$$\text{Bacteriocin}\;\text{activity}=\left(mm^{2}/ml\right)={\text{L}}_{\text{z}}-{\text{L}}_{\text{s}}/\text{V}$$

- L_z_=clear zone area (*mm*)^2^
- L_s_=well area (*mm*)^2^
- V = volume of sample (*ml*)

### Identification of DDBCC70

The bacteriocin-like producer candidate, DDBCC-70, was morphologically and biochemically characterized according to the standard procedures in Bergey’s Manual of Systematic Bacteriology ^22^. 16s rDNA gene was amplified by 27F; 5′-AGAGTTTGATCCTGGCT CAG and 1492R; 5′-CGGTTACCTTGT TACTT primers ^23^ under a program for 3 *min* at 94°*C*, 30 cycles of 1 *min* at 94°*C*, 1 *min* at 52°*C* and 2 *min* at 72°*C*, followed by 72°*C* for 10 *min*. The amplicons were sequenced (Bioneer Company) and analyzed. DDBCC70 is a member of Dasht-Desert Bacterial Culture Collection (http://www.wfcc.info/ccinfo/collection/by_id/1185).

### Statistical analyses

The data were tested for normality and then analyzed for the traits using analysis of variance (ANOVA) and Duncan’s multiple range test (p<0.05) in SPSS version 22.

## Results

### Screening DDBCC against the indicators

Out of 170 isolates, 17 strains showed an antibacterial effect against *B. subtilis*, while 72 strains inhibited *Proteus* (spp.). The growth of *P. aeruginosa* was limited by 55*, S. aureus* by 55, *E. coli* by 57 and *Salmonella* (spp.) by 57 isolates. Twenty-three isolates with antibacterial activity against at least three indicators (broad spectrum) were further analyzed.

### Well diffusion assay

Next, 23 strains were tested for their antibacterial activity using well diffusion agar method. The largest and the smallest inhibition zone were detected against *K. pneumoniae* and *B. cereus*, respectively. *S. aureus*, *Salmonella* (spp.), *B. subtilis* and *Proteus* (spp.) were inhibited by 9, 8 and 7 members of DDBCC. *E. coli* was susceptible to 5 and *P. aeruginosa* to 2 DDBCC isolates. Among DDBCC isolates with an inhibition zone of about 12 *mm*, DDBCC70 harbored the widest antibacterial spectrum (p<0.01) against G^+^ and G^−^ bacteria. Applying the CFS of DDBCC70, no inhibition zone was detected against *Proteus* (spp).

### Bactericidal assay

**Figure 1.**
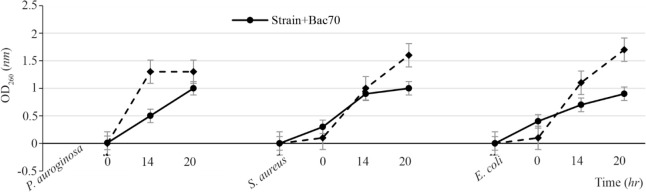
Antimicrobial effects of CFS from DDBCC70 against indicator bacteria; *E. coli*, *S. aureus* and *P. aeruginosa* (p<0.01)

The supernatant from DDBCC70 decreased the growth rate of the indicators by 50%. The significant inhibitory effect was detected against *S. aureus, E. coli* (50%) and *P. aeruginosa* (30%). The CFUs decreased 6 fold within 2 *hr*. Figure 1 (p<0.01) displays the growth rates of the indicators under the stress of CFS from DDBCC70.

### Optimization of physicochemical condition

The effects of physical parameters on the function of Bac70 showed stability in a broad range of pH (3–11) (Supplementary 1) and temperature (−80 to 100°*C*), although the temperature of 121°*C* and UV-irradiation obviously reduced its antibacterial activity. Bac70 was stable under the stresses of inorganic salts (Supplementary 2), and detergents (Supplementary 3). CdCl_2_ (5 *mM*) showed a synergistic increase to 65% in the antibacterial activity (p<0.01). Ethyl-acetate and methanol decreased the activity of Bac70 by 15%. Diethyl ether, ethanol, and chloroform did not influence the bacteriocin-like activity, while it rose synergistically with butanol (31.9%), dichloromethane (14%), isopropanol (16%), and acetone (28%). Enzymatic treatment with proteinase K decreased the activity of Bac70, confirming its nature as a peptide.

**Supplementary 1.**
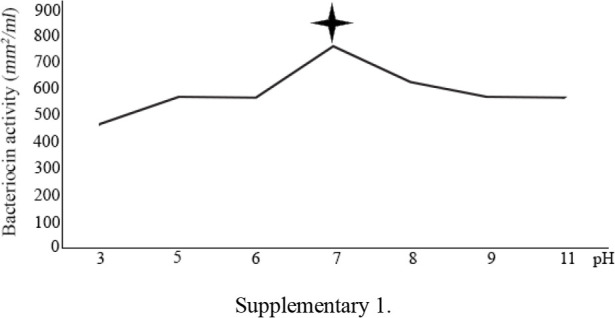


**Supplementary 2.**
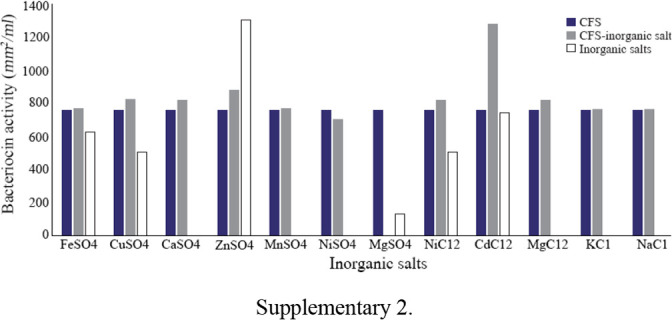


**Supplementary 3.**
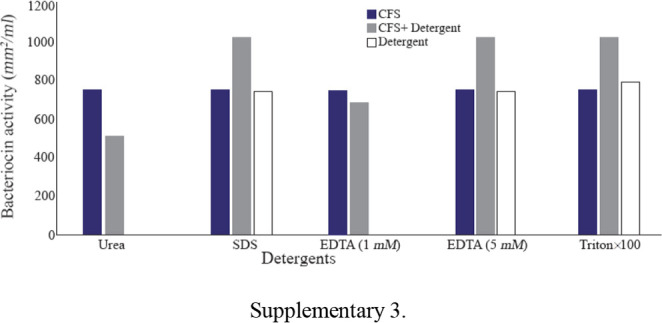


**Figure 2.**
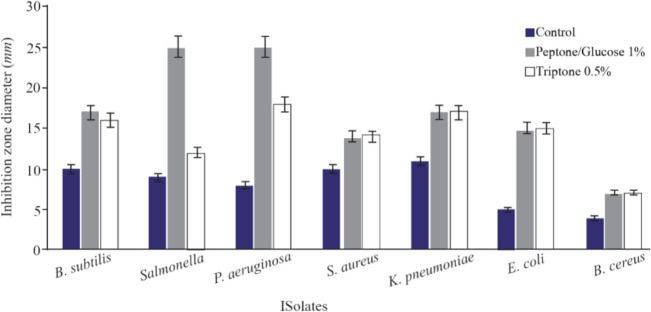
Optimization of carbon and nitrogen resources for producing bacteriocin; (p<0.01).

**Figure 3.**
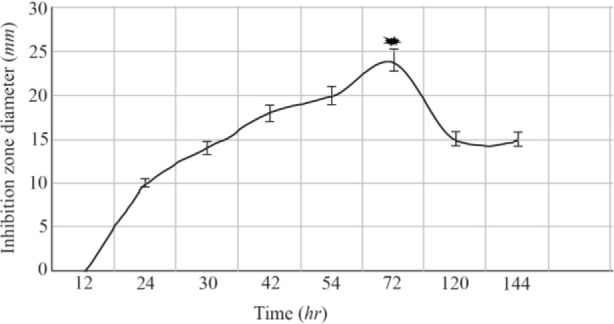
The kinetics of bacteriocin production (p<0.01).

The optimization experiments for nitrogen resources in media led to the greatest zone of inhibition against *B. subtilis*, *E. coli* and *S. aureus* in the BHI supplemented with 1% peptone or 0.5% tryptone (*w/v*) (Figure 2). The optimization of carbon resources using M9 medium produced 5 *mm* inhibition zone diameter adding 1% glucose and/or mannitol. Similar results were obtained using BHI modified by 1% glucose and 1% peptone (*w/v* carbon and nitrogen resources) as the optimum medium for the production of Bac70 in DDBCC70 (p<0.01; Figure 2). DDBCC70 produced the highest amount of Bac70 in the modified BHI at 37°*C* and pH=7 in 72 *hr*, where the maximum zone of inhibition was detected against *K. pneumonia*, *S. aureus* and *B. cerues* (p<0.01; Figure 3).

### Partial purification of bacteriocin-like Bac70

The purified Bac70 (70% saturation) showed the highest antibacterial activity against *S. aureus*, *K. pneumoniae*, and *B. cereus*. The pure Bac70 killed *P. aeruginosa*, while the CFS only inhibited its growth.

**Figure 4.**
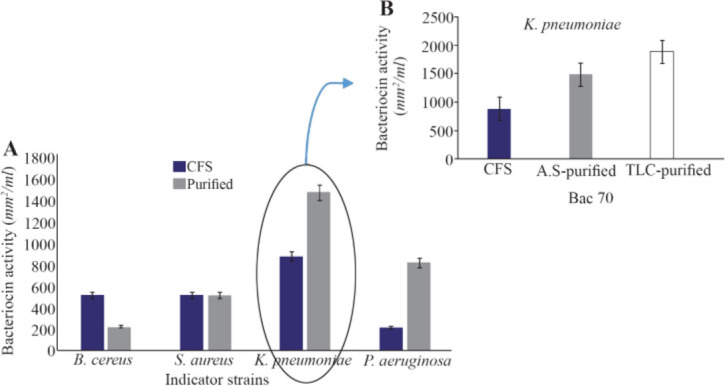
A) Purification of bacteriocin using ammonium sulfate (p<0.01), B) The activity of Bac70 in cell-free supernatant (883 *AU/ml*), purified with ammonium sulfate (1479 *AU/ml*), and purified fraction (RF 0.64) from TLC (1885 *AU/ml*) against *K. pneumonia.*

The activity of purified Bac70 on *P. aeruginosa* was 3.8 fold of the CFS. No reliable difference in the growth of *B. cereus* and *S. aureus* was detected under the stress of purified Bac70 (p<0.01; Figure 4A). The highest activity of purified Bac70 was found as 1479 *AU/ml* against *K. pneumonia*e (Figure 4B) with a 1.67 fold increase in the non-purified one.

**Figure 5.**
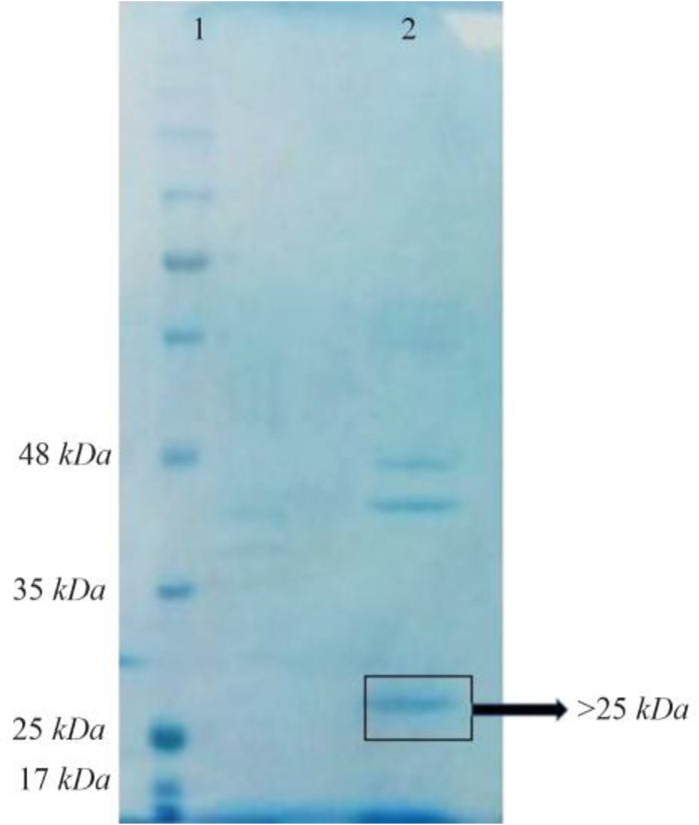
SDS-PAGE of purified-bacteriocin from DDBCC70; 1: weight maker, 2: Bac70 after ammonium sulfate precipitation. The dashed circle shows the Bac70 peptide.

Bac70 as a >25 *kDa* protein on SDS-PAGE is shown in figure 5. The amount of Bac70 in the CFS and purified form were determined as 200 and 1000 *μg/ml*, respectively. The MIC and MBC of the purified Bac70 against *K. pneumonia*e were 40 *AU/ml* and 1280 *AU/ml*, respectively (p<0.01).

### Qualitative analysis of Bac70

Visualizing the TLC plate by ninhydrin staining led to red and pink spots indicating the nature of peptide for Bac70. The inhibition zone of the TLC-purified fraction (RF 0.64) against *K. pneumoniae* was 3 *mm* larger than that of purified Bac70 (p<0.01; Figure 4B).

### Cytotoxicity assays, hemagglutination, and hemolytic activity

Bac70 was found a safe compound for blood cells in the MTT test (p<0.01). Furthermore, no hemolytic activity was detected on blood agar.

### Biofilm disruption by Bac70

The purified Bac70 showed strong inhibitory effects against biofilm formation of *P. aeruginosa* and *S. aureus*. Bac70 destroyed 30.33% and 66.66% of the bio-films formed by *P. aeruginosa*, and *S. aureus*, respectively (p<0.01).

### Bac70 andpenicillin synergy

In a synergy test on Bac70 and penicillin G-resistant *Proteus* (spp.), penicillin G created a clear zone of 20 *mm* along with Bac70.

### Identification of DDBCC70

The black pigments on BHI agar plus a 98% query cover in 16S rDNA sequence (BLAST) and similarity in biochemical tests (Table 1) confirmed the strain of DDBCC70 as *Bacillus atrophaeus* (Gene Bank: MH-685187.1).

**Table 1.** Biochemical identification of DDBCC70 in comparison to *B. atrophaeus*

**Biochemical characterization**	***B. atrophaeus*** ^*^	**DDBCC70**
**Pigment color**	Brown	Brown
**Gram stain**	+	+
**Spore production**	+	+
**Bacterial morphology**	Rod-shaped	Rod-shaped
**Catalase**	+	+
**Oxidase**	+	+
**Capsulated**	+	+
**Simmons’ citrate**	+	+
**Starch hydrolysis**	-	-
**Motility**	+	+
**Indole production**	-	-
**Hydrogen sulfide on TSI**	-	-
**Methyl red**	-	+
**Voges proskauer**	+	-

* [14]

## Discussion

The potential of Dasht-Desert (Semnan province) microbiota for producing bacteriocins or bacteriocin-like molecules was discussed here. Based on the literature, bacterial culturable isolates from desert ecotypes exhibit metabolic activity in a wide range of physicochemical influences. A study on soil bacterial communities of Sahara and Gibson deserts showed the functional differentiation leading to bacterial sustainability and moderate biodiversity. The morphological and physiological variation of the isolates resulted in a physiological adaptation of the microbial communities in continual change of environmental conditions of desert followed by increasing their resistance. Production of antagonistic molecules from bacteria may have a key role in reducing the biodiversity in microbial habitat ^24^.

Similar to other studies ^25,26^, 54% of isolates from DDBCC showed antibacterial activity against at least one indicator bacterium. Among the isolates from DDBCC, DDBCC70 (*B. atrophaeus)* harbored antibacterial activity against *B. cereus*, *B. subtilis*, *P. aeruginosa*, *Salmonella* (spp.), *K. pneumoniae, E. coli,* and *S. aureus*.

Based on the following observations, the antibacterial agent from DDBCC70 was concluded to be a bacteriocin or bacteriocin-like protein. The pH value of supernatant from DDBCC70 was 7, approving that no efficient acidic metabolite(s) killed the tested bacteria. The negative results from blood hemolysis experiments showed that the antibacterial agent(s) was not a surfactant. The antibacterial agent from DDBCC70 was susceptible against proteinase K and behaved like a peptide under physical stresses. Thin layer chromatography analyses revealed the nature of antibacterial agent from DDBCC70 as a peptide. The peptide band eluted from TLC plate displayed antibacterial activities confirming that the compound is a bacteriocin or bacteriocin-like molecule. The >25 *kDa* protein was found on the acrylamide gel electrophoresis regarding the Bac-70.

The components of the culture medium affect the biosynthesis of secondary metabolites both quantitatively and qualitatively ^12^. The role of carbon and nitrogen resources plus pH and temperature in the biosynthesis of bacteriocins has been well investigated. The maximum yield of KIBGE IB-17 from *B. subtilis* was reported in the medium containing 1.0% tryptone, 0.5% yeast extract and 0.5% NaCl at 37°*C* after 24 *hr* and pH=7.0 ^27^. The production of BACYAS1 was optimized in the peptone yeast beef medium (0.48% yeast extract) in 62 *hr* at 37°*C* ^28^. DDBCC70 produced the highest amount of bacteriocin-like activity in the modified BHI (1% glucose and peptone). Absorption of bacteriocin on the producer cells and/or proteolytic degradation ^29^ might cause a significant reduction of antibacterial activity after 72 *hr*.

Bac70 was stable in the pH of 3–11 and temperature of −80 to 100°*C*, although the temperature of 121°*C* and UV-irradiation reduced its antibacterial activity. H4 bacteriocin was stable under the stresses of 60–100°*C* and pH of 2–10 ^30^. Heat and pH stability of CAMT2 ^31^ were nearly the same as Bac70 with a noticeable advantage for Bac70 with a wider range of heat tolerance. Gupta *et al* introduced the strain BP03 with antimicrobial activity against pathogens at high temperature (121°*C*), high salt concentration (10% *w/v*) and over the wide range of pH (3, 6, 7, 9) ^32^. The antibacterial activity of bacteriocin against Gram positive bacteria indicator *S. aureus* followed by *E. coli* and *Salmonella* (sp) at pH=7, 37°*C* and storage at 4°*C* was shown by Hussain SN ^33^.

Bacteriocins are substrates for trypsin and proteinase K ^10^. These evidences confirmed the proteinaceous characteristics of the antibacterial agent. Enzymatic treatment with proteinase K decreased the activity of Bac70 confirming its proteinaceous nature.

Here, ammonium sulfate was used to precipitate BAC70. This method as a ‘salting-out’ method has been widely applied for protein and peptide separation based on their solubility under increasing ionic strength. The ammonium sulfate precipitation method is a conventional, inexpensive and laborious method for bacteriocin purification. As it was reviewed, the bacteriocin concentrate obtained from ammonium sulfate method exhibits an increase in the inhibition zone diameter against tested bacteria. Our results were in agreement with this document ^34,35^. The rise in the activity of purified Bac70 in comparison to non-purified CFS revealed that the antibacterial agent from DDBCC70 was most probably a bacteriocin or bacteriocin-like molecule because of its specific function. The MIC of purified Bac70 was 40 *AU/ml* against *K. pneumonia*e, where the obtained MBC was 1280 *AU/ml*. Under the optimum conditions, BACYAS1 showed an activity of 470 *AU/ml* against *E. amylovora* ^28^. In the purification steps of a 21 *kDa* bacteriocin from *Lactococcus lactis* JC10, the activity units increased to 9500 *AU/ml* against the indicators ^36^.

The bacteriocin peptides are known to be noncytotoxic, although some bacteriocins have been reported with significant toxic effects on macrophage cell lines and ASCs ^18,37^. Based on the cytotoxic test, Bac70 was a safe compound for blood cells.

Biofilm formation on hospital devices by *S. aureus* and *P. aeruginosa* has been a global problem. Bacteriocins isolated from *Lactobacillus plantarum* showed inhibitory effects on biofilm formation and antibacterial activity against *S. aureus, S. sanguinis,* and *P. aeruginosa* ^8^. Sonorensin, a bacteriocin from *B. sonorensis*, inhibited biofilm formation of *S. aureus* ^38^. The bacteriocin isolated from *B. subtilis* reduced biofilm formation by 88% and disrupted mature biofilms by 81% ^39^. In the current study, purified Bac70 destroyed 66.66% of biofilm of *S. aureus,* and 30.33% of *P. aeruginosa*. Mathur H *et al* reported the role of bovicin HC5 and nisin in reducing cell adhesion via the expression of biofilm-associated genes (Quorum sensing) and altering the hydrophobicity of the cell surface ^40^. Okuda *et al* evaluated the influence of three bacteriocins including nisin A, lacticin Q, and nukacin ISK against planktonic cells and biofilm cells. Among the bacteriocins, nisin and lacticin Q showed the highest bactericidal activity against both planktonic cells and biofilm cells ^41^.

## Conclusion

Here, Bac70 is introduced with significant antibacterial and anti-biofilm activities. The main features of the bacteriocin from DDBCC70 are specific inhibitory effects against *P. aeruginosa* and *K. pneumoniae*, disruption of biofilms from *S. aureus* and *P. aeruginosa*, no cytotoxicity effects, no hemagglutination and hemolytic activities, pH and temperature stability, and low susceptibility to organic and inorganic chemicals. Considering biofilm disruption as a rare feature of bacteriocin-like compounds, the inhibitory effect of Bac70 against biofilms is a compelling advantage. Based on the features, Bac70 is a potential alternative therapeutic agent in pharmaceutical, food preservative, and biotech-related industries.
